# Partial Sets of Teeth

**Published:** 1850-07

**Authors:** W. H. King

**Affiliations:** Pittsburgh, Pa.


DENTAL REGISTER OF THE WEST.

VOL. III. JULY, 1850. NO. 4.

### PARTIAL SETS OF TEETH.

### A TREATISE ON THE METHOD OF CONSTRUCTING PARTIAL SETS OF TEETH; SUBMITTED TO THE FACULTY OF THE OHIO COLLEGE OF DENTAL SURGERY, AT THE CLOSE OF THE SESSION OF 1849 AND 1850.

BY W. H. KING, OF PITTSBURGH, PA.

As you, the honorable Faculty of the Ohio College of Dental Surgeons, have made it obligatory upon the Dental Student, wishing to present himself as a candidate for graduation, to select and present his views to your consideration, on some subject pertaining to Dental Science, I, for that purpose, have selected one of the specialities of mechanical dentistry, viz.: The insertion of partial sets of teeth on plate.

It is my design first to refer very briefly to the preparation of the mouth, necessary in many cases, preparatory to the insertion of artificial work. Then, of the materials used, and the method of using them.

The condition of the mouth cannot be too closely observed, in the application of artificial teeth. To the neglect of this, many of the evil effects, resulting from their use, are attributable.

No artificial apparatus, no matter how correct in its construction, can be worn with comfort in a diseased mouth.

The mechanical and chemical bearing alone, of artificial teeth on diseased gums, would naturally become an additional aggravating cause of disease. The patient, moreover, would find it impossible to preserve the cleanliness of his mouth; and his artificial apparatus, in combination with the diseases of the mouth, would become a source of pain and trouble, and the whole mouth would be rendered offensive to himself, as well as to others.

The first thing, then, claiming the attention of the dentist, when applied to for artificial teeth, is to ascertain the condition of the gums and such teeth as are remaining: and if diseased, he should at once adopt such treatment as the circumstances of the case indicate.

He should extract all old roots, and such teeth as cannot be preserved by filling—then cleanse and fill such teeth as are diseased. In short, nothing should be left undone that would in any way benefit the condition of the mouth.

After being fully satisfied that all is done that can be done to prepare the mouth for the reception of artificial teeth, a sufficient length of time should be allowed for the absorption of the alveoli, or, otherwise, insert a temporary operation, which in some cases, can be done immediately after extracting; in others, it is necessary to wait two or three weeks, depending altogether on the condition of the mouth.

A temporary operation can be worn, as a general thing, sufficiently long for complete absorption to take place, but not without some inconvenience, from the fact of the plate losing its adaptation, by the absorption of the alveoli.

The first thing to be done in the construction of artificial teeth on plate, is to procure a correct impression of the part upon which the plate is to rest, and on the correctness of the impression depends the whole operation. For this purpose, there have been a number of materials made use of—the different preparations of wax, plaster of paris, gutta purcha,—and perhaps no one without some objections.

The objections to the wax are, that it may be sometimes slightly misplaced in loosening from the gum, and with drawing from the mouth. Both of these objections, however, can be generally obviated, by proper shape of cup, and not using too much wax, so as to force it without the rim of the cup, and be thus subject to the pressure of the lips, in removal from the mouth. The lips, however, should be held as much out of the way as possible.

The difficulty of loosening a good impression from the gum, so as to avoid the displacement of the wax, can be obviated by having one, two, three or more holes punched in the cup, so that, after the wax has been forced to the gum, and so accurately pressed to every part, that the air is completely exhausted, leaving a perfect suction, small probes may be introduced through these holes and wax till the gum is touched, thus letting in the air, so that the impression will come away without the application of any force. The pure bees-wax, or that which is called adhesive, thus used, will always ensure a good impression. In soft gums, where temporary teeth are to be inserted, the wax should be correspondingly soft; yet, in all cases, it should be soft enough, so as not to require much force in pressing it to every part of the gum.

The objections to the use of plaster-paris, are, first, the difficulty many persons experience in retaining it sufficiently long in the mouth to harden—the disposition, in many cases, to gag, being such, that expedition is even requisite in the use of wax. The second difficulty is, that when teeth remain in the mouth, their necks being often smaller than their crowns, the plaster, when hardened, cannot be removed without displacement of the same around these teeth. These objections may, it is true, most generally be overcome—the necks of the teeth may be enlarged by the use of adhesive wax, and many persons are not gagged by a mouth ever so full. The plaster should be about the consistence of thick cream, mixed up with pure water, with a little salt, which facilitates the hardening, and held firmly, but gently, to its place till perfectly set, or so hard as not to be injured by removal from the mouth.

Impressions thus obtained may always be correct, yet, perhaps, not more so than with wax, and as it is more tedious and unpleasant to the patient, I see no propriety in discarding the wax for its use.

The objections to the use of gutta percha are, first, the heat which is requisite to make it the proper consistence: this being greater than can often be well borne in the mouth—and second, the contraction which this substance undergoes in cooling. These render this article objectionable, and I believe that the Profession have generally been disappointed in their expectations of it.

In taking an impression with wax, it is necessary to heat it in warm water, or by the fire, until it is about the consistence of dough or putty; it should then be placed in a frame, made for the purpose, and sufficiently large to embrace the part upon which the plate is to rest, and also, the teeth to be embraced by the clasps.

It should then be placed in the mouth, and pressed firmly against the part to be filled with artificial substitutes—continue the pressure, until the teeth and gums are completely embedded in the wax.

The finger should then be passed round, pressing the wax against the labial surface of the gums, and also, in the central portion of the palate.

The frame should now be slightly tilted, so as loosen the wax, and then carefully withdrawn, taking care not to disturb, or drag the wax, ortherwise, the operation will have to be repeated.

Having obtained the impression, the next process is to procure a model from the wax with plaster of paris. Before doing this, however, the impression in the wax should have a light coat of oil, to prevent its sticking to the plaster, and also, a small tack stuck in each impression in the wax, made by the natural teeth, for the purpose of strengthening the teeth on the plaster model.

There should then be placed around it, a waxed cloth, forming a cup, near the shape the cast should be, to withdraw easily from the sand, when moulding.

A portion of the best calcined plaster of paris, should now be mixed with water, until it acquires the consistence of cream, then gently pour a little of it on an elevated portion of the impression, and allow it to run into the holes, made by the teeth, in the wax; the object of which, is to prevent air-bubbles in the plaster cast, which would occur, if the plaster was poured in quickly. When the impression is filled on a level with its edges, continue to add plaster until it is an inch or two in heighth. When hardened, it will be sufficiently strong to withstand all the pressure necessary, in taking a metalic model.

In a few minutes, if the plaster used is good, it will be sufficiently set, to allow the separation of the wax from the plaster: this is best done, by heating the plaster model on a stove, or on a piece of sheet iron placed over the fire, until the wax becomes soft, when it can be removed with ease and safety.

The plaster model should now be trimmed to a proper shape, that it may be readily withdrawn from the sand when taking a metalic cast.

It should then be varnished, and thoroughly dryed, which will give it a surface that will prevent the model wearing away by use, and also, facilitate its removal from the sand.

Having thus procured the plaster model, it is necessary to obtain a metalic model, and counter model, in some hard metal, by which a gold plate may ultimately be formed. For this purpose, there have been a number of metals made use of—copper, brass, zinc, tin, lead, and bismuth in their various combinations.

But, among all the metals yet brought into use, for this purpose, pure zinc, or zinc alloyed with tin, in the proportion of two thirds zinc and one third tin, is, perhaps, preferable for the male cast, from the fact, that it can be fused in an ordinary fire, and is also, sufficiently hard and firm for all purposes.

For the matrix or counter model, lead is preferable, for ordinary purposes, for the same reason—that it is easily fused—and it being so much softer than the zinc, it is not liable to stretch the gold plate in swedging, which would occur often, if both metals used were equally hard.

There are some cases, however, in which it may be necessary to use a harder metal for the counter model, particularly where the ruga of the mouth is very prominent. In such cases, tin should be used, and when used, it should be hammered as little as possible, otherwise it is liable to stretch the plate.

There are are several methods of obtaining a metalic model and counter model of these. I will describe but one. It consists in filling a box, four or five inches wide, and five or six in depth, with sand, such as is used in brass and iron founderies, previously moistened with water, to a consistence that will allow it to adhere when pressed.

The plaster model is now pressed firmly into the sand, then gently tapped with a light hammer, for the purpose of detaching it from the sand, and carefully removing it. This should be repeated, if necessary, several times, pressing a little harder each time, until a correct impression, as far as possible, is obtained.

The mould now in the sand should be filled with zinc, previously melted in a common iron ladle.

When cooked, any imperfections around the necks of the teeth, can be removed by a file or an engraver, and made to represent the plaster model.

To procure this counter model, fill the box with sand, as before, and place the zinc cast in it, with the part presenting a transcript of the plaster model upwards, while the remaining part should be buried in the sand. The cast, thus placed, should be encircled with a piece of sheet iron, three or four inches wide, and sufficiently long to lap two or three inches at the ends, when bent to a circle. The lead or tin, previously melted, should now be poured on it. When the metals have cooled, the castings may be separated with a hammer, and, if perfect, are ready for use.

Having proceeded thus far, the next step in the operation is, to procure a base, upon which the artficial teeth are to set. For this purpose, gold should be used, as it is the only metal we have that can readily be reduced to the proper form, and which the secretions of the mouth will not act upon. In using gold, then, for this purpose, it should never be less than eighteen carats fine,* and as much finer as the case will allow, so as to have sufficient elasticity.

Being now provided with the castings and gold plate, proceed to cut a piece of sheet lead, by the zinc cast, the shape and size the gold plate is intended to be; this place on the sheet gold, and with a point trace its outline, then, with a pair of snips and file, it can be cut to the proper shape.

The plate thus formed, should be placed on the zinc, and by means of a small wooden hammer, adjusted as nearly as possible to its intended form. The plate should then be annealed and placed in the counter model, and with a heavy hammer struck up: this should be repeated, until the gold is forced into the various inequalities of the metalic cast.

The plate should now be applied to the plaster model, for the purpose of adapting the clasps; and if that portion of the plate coming in contact with the natural teeth remaining in the mouth should press against them, it should be filed away, and also, around the teeth to which the clasps are to be attached, allowing for their additional thickness.

The clasps should now be accurately fitted to the teeth, leaving them as wide as the crown will allow, to prevent any unnecessary friction on any one part of it by the clasps.

When accurately fitted to the teeth and plate, they should be retained in their position by means of wire, and soldered fast. This is done by covering the edges of the two pieces to be united with borax, previously prepared by grinding the borax, with a little water, on a piece of glass or slate, until it acquires about the consistence of cream. After which, a few pieces of solder should be placed along the line of connection, between the clasp and plate, and heat applied until it flows.†

---

* We think that gold should never be less than twenty carats fine. This is our usual quality.—J. T., Cin. Ed.

† It is well to state here that Dr. King describes the usual method of this

The plate should now be removed from plaster model, and thrown into a mixture, of equal parts of sulphuric acid and water, for the purpose of dissolving the borax that will be found sticking to it—this will require but a few minutes. It should then be taken from the acid, and the solder made smooth by suitable files and scrapers, when it is ready to try in the mouth.

The plate now being fitted to the mouth, the next thing is to ascertain the size and length of the teeth, necessary to fill the vacant space: this is done by placing wax on the part of the plate to which the teeth are to be applied, and placing it in the mouth, directing the patient to close the jaws, naturally and closely together, imbedding the teeth of the opposite jaw in the wax—now mark on the wax the length of the teeth. This will give the length of the teeth to be used, and also, the arch of the antagonizing teeth, and the point at which each one strikes. The plate should now be removed from the mouth, and placed on the plaster model, for the purpose of selecting and grinding the teeth.

If the space to be filled does not exceed the space occupied by six or eight teeth, it will not be necessary to take an articulating model; for the teeth on the plaster model, with the bite in the wax, is a sufficient guide, by which the teeth can be fitted to the plate, so as to articulate in the mouth.

But if the operation be eight or ten teeth, or all in the one jaw, it will then be necessary to have an articulating model, as in full sets of teeth.

When the space to be filled only requires six or seven, it is important that the artificial ones correspond, in color, with the natural teeth, for in proportion as they are darker or whiter will be the contrast, and liability of detection increased.

As they are selected, they should be arranged on the plate, and retained in their place by the wax already on it. If they

---

part of the operation, or that which we generally practice, but that occasionally all the various plans suggested by Prof. Harris and others, who have written more recently on this subject, are resorted to—the object being always to get a perfect adaptation of clasps, and an easy retention of the plate in the mouth.—J. T., Cin. Ed.

do not fit closely to the plate and gum, they should be ground on an emery wheel or small grind-stone until they do, and also, antagonize with the teeth of the opposite jaw, at the same time.

The teeth, after being thus arranged and adjusted in the mouth, should be placed in a cast iron frame, with a mixture, of equal parts plaster of paris and sand, and allowed to harden. The wax should now be removed, and the teeth lined without removing them from their place. The lining of the teeth should be accurately fitted to the backs of the teeth, and also to the plate; the platina pins should then be split with the point of a knife, to keep the linings in their place until soldered.

This done, borax triturated in water, as before described, should be applied with a camels hair, to the part where it is wished that the solder should take effect. After applying the borax, a number of small pieces of solder should be applied immediately on the line of connection, between each tooth and the plate, and on each rivet. It should now be placed in the fire, and allowed to remain until it is raised to a red heat; it should then be taken out and put under the blaze of the blow pipe, throwing the blaze directly on the part where the solder is designed to flow, until the operation is completed.

When cooled, it should be removed from the plaster and sand, and put into acid and water as before, to cleanse the surface.

The piece should then be washed, and any rough portions of solder carefully removed by means of scrapers and files. After it has been made as smooth as possible in this way, it should be rubbed with Scotch-stone and water.

It is then ready for finishing with rotten stone, rouge and brush—and lastly, for boiling in the mixture recommended in the last edition of Dr. Harris's work.

